# Bioactivity of Heat-Processed Korean *Curcuma longa* is Preserved Despite Extensive Curcuminoid Degradation

**DOI:** 10.4014/jmb.2606.06010

**Published:** 2026-09-18

**Authors:** Choong-In Yun, Ye Seol Shin, Min Jeong Kim, Sung Keun Jung, Young-Jun Kim

**Affiliations:** 1 Department of Food Science and Biotechnology, Sungkyunkwan University, Suwon 16419, Republic of Korea; 2 School of Food Science and Biotechnology, Kyungpook National University, Daegu 41566, Republic of Korea; 3 Research Institute of Tailored Food Technology, Kyungpook National University, Daegu 41566, Republic of Korea; 4 Department of Food Science and Biotechnology, Seoul National University of Science and Technology, Seoul 01811, Republic of Korea; 5 Research Institute of Food and Biotechnology, Seoul National University of Science and Technology, Seoul 01811, Republic of Korea

**Keywords:** *Curcuma longa*, *Ulgeum*, Thermal processing, Anti-inflammatory, Antioxidant, Phytochemical

## Abstract

*Ulgeum* is a Korean-cultivated variety of *Curcuma longa* L. that is commonly consumed after heat processing, such as drying or decoction. Although the bioactivity of unheated or mildly heated *C. longa* extracts (CLEs) has been widely investigated, the functional consequences of high-temperature processing remain poorly understood. Heat treatment at 180°C markedly reduced curcuminoid levels, while sesquiterpenoids, such as *ar*-turmerone, exhibited comparatively greater thermal resistance, and these changes were accompanied by the formation of low-molecular-weight phenolic degradation products. Despite these compositional shifts, heat-treated CLEs retained substantial biological activity. In LPS-induced RAW 264.7 macrophages, CLEs suppressed nitric oxide production through the selective attenuation of NF-κB signaling, without significantly affecting MAPK pathways. In parallel, antioxidant activity was preserved across heat-treatment durations up to 90 min, as evidenced by a sustained suppression of intracellular reactive oxygen species production and activation of Nrf2-dependent antioxidant responses. Collectively, these findings demonstrate that the bioactivity of *ulgeum* is resilient to high-temperature processing and is maintained through a coordinated modulation of inflammatory and redox signaling pathways rather than through the preservation of curcuminoids alone.

## Introduction

*Curcuma longa* L. (Zingiberaceae) is a spice crop widely cultivated in tropical and subtropical regions that has long been a source of antioxidant and anti-inflammatory compounds in food and utilized as a traditional medicine [1, 2]. Korean-cultivated *C. longa*, commonly referred to as *ulgeum*, exhibits distinct underground growth characteristics, including the development of both rhizomes and tuberous roots under Korean cultivation conditions [3, 4]. These ecological and morphological differences influence the relative abundances of curcuminoids and sesquiterpenoids, reflecting the metabolic diversity among geographically distinct *C. longa* populations [5]. Despite its long history of use in Korean traditional medicine, *ulgeum* has received far less scientific attention than tropical *C. longa* varieties, and its biochemical and molecular properties remain insufficiently characterized.

The principal bioactive constituents of *C. longa* include curcuminoids—curcumin, demethoxycurcumin (DMC), and bisdemethoxycurcumin (BDMC)—as well as sesquiterpenoids, such as *ar*-turmerone and bisacurone [4, 6, 7]. Curcuminoids exhibit a broad spectrum of biological activities, including antioxidant, anti-inflammatory, and anticancer effects [1, 8, 9]. However, they are highly sensitive to heat, light, and oxygen, and therefore prone to rapid degradation during food processing or cooking. Curcumin, which contains a β-diketone moiety, undergoes oxidative cleavage, molecular rearrangement, and condensation at elevated temperatures, leading to a sharp decline in its concentration during heating [5, 10]. Nevertheless, recent studies have shown that the thermal degradation of curcuminoids does not necessarily result in a complete loss of bioactivity [8, 11]. During heating, several low-molecular-weight degradation products, including vanillin, dehydrozingerone, and ferulic acid, are formed [7, 11, 12]. By contrast, sesquiterpenoids are structurally more stable and exhibit a higher resistance to thermal decomposition than curcuminoids [4, 13]. Thus, thermal processing appears to alter, rather than completely transform, *C. longa*’s chemical profile. It is important to note that these thermal behaviors have been characterized primarily in other *C. longa* varieties, and it remains unclear whether *ulgeum* exhibits comparable degradation and stability patterns during heating. Comprehensive analyses correlating heat-induced compositional changes with molecular and functional bioactivities remain limited. Since *ulgeum* is a common component of cooked or processed foods, understanding how heating alters its chemical and functional properties is essential for evaluating its physiological relevance.

In this study, we evaluated the anti-inflammatory and cytoprotective effects of *ulgeum* using two complementary experimental models. Lipopolysaccharide-induced macrophage activation was used to assess inflammatory responses, and exposure to 1-nitropyrene (1-NP), a nitro-polycyclic aromatic hydrocarbon and a major constituent of particulate matter [14, 15], was used to evaluate pollutant-induced cytotoxicity. Given the increasing health burden associated with airborne pollutants [16], identifying natural products capable of mitigating pollutant-induced toxicity has become an important research priority. Our previous work demonstrated that 1-NP is cytotoxic to lung epithelial cells, highlighting its relevance as a pollutant-associated stressor [17]. Although thermal processing can modulate the bioactive potential of plant-derived phytochemicals, no study has yet examined whether heat-induced chemical changes in *ulgeum* alter its ability to modulate LPS-driven inflammation or influence cellular resilience to pollutant-induced injury. As thermal effects are often time dependent, variations in heating duration may differentially modify the stability of *ulgeum* constituents or transformation, potentially giving rise to distinct biological outcomes. By using complementary macrophage and epithelial cell models, this study aimed to characterize the compositional and molecular alterations induced by heat treatments in *ulgeum* and evaluate its anti-inflammatory and cytoprotective activities under distinct experimental conditions.

## Materials and Methods

### Reagents

Curcumin, demethoxycurcumin (DMC), bisdemethoxycurcumin (BDMC), and vanillin-^13^C_6_ were obtained from Toronto Research Chemicals (Canada). Vanillin, bisacurone, *ar*-turmerone, and vanillylidenacetone (dehydrozingerone) were purchased from Sigma-Aldrich (USA). Formic acid and acetic acid of LC–MS grade were also supplied by Sigma-Aldrich. High-purity solvents, including acetonitrile, absolute ethanol, and water suitable for HPLC and UHPLC–MS/MS analyses, were sourced from J.T. Baker (USA).

### Materials

Dried *Curcuma longa* (*Ulgeum*) rhizomes, cultivated in the Republic of Korea during the 2023 growing season, were obtained from a licensed supplier, CK Pharm Co., Ltd. (Republic of Korea). The company provided documentation certifying that the raw material met standard requirements for safety and quality, including the morphological confirmation of plant identity. To genetically verify the botanical origin, powdered rhizomes were subjected to DNA extraction using the cetyltrimethylammonium bromide (CTAB) protocol described by Chen *et al*.[18]. Two DNA barcode regions—ITS2, from the nuclear genome, and trnK/matK, from the chloroplast genome—were amplified using primer sets ITS2ZF/ITS8ZR and trnK3914F/CT828R, respectively [4, 18]. The resulting PCR amplicons were sequenced using the Sanger method, and the resulting sequences were searched against the GenBank database (https://blast.ncbi.nlm.nih.gov/Blast.cgi) using BLASTn, revealing a high degree of similarity to *C. longa* reference entries (*e.g.*, MW838984.1 and AB047738.1), thereby confirming the taxonomic authenticity of the rhizome material.

Dulbecco’s modified Eagle’s medium (DMEM), antibiotics (penicillin–streptomycin), and fetal bovine serum (FBS) were purchased from Thermo Scientific Inc. (USA). *Escherichia coli* O111: B4, phosphoric acid, sulfanilamide, and thiazolyl blue tetrazolium bromide were obtained from Sigma-Aldrich Co., N-(1-naphthyl) ethylenediamine dihydrochloride and 1-NP was purchased from TCI Chemicals (TCI Chemicals, Japan). Primary antibodies against iNOS (#13120, 1:1000), COX-2 (#12282, 1:1000), p-p65 (#3033, 1:1000), p65 (#8242, 1:1000), p-IκBα (#2859, 1:1000), IκBα (#4814, 1:1000), p-IKKα/β (#2697, 1:1000), IKKα (#61294, 1:1000) were purchased from Cell Signaling Technologies (USA). Primary antibodies against β-actin were purchased from Santa Cruz Biotechnology, Inc. (USA).

### Sample Preparation and Heat Treatment

The dried rhizomes of *C. longa* were ground using a mixer and sieved through a 50-mesh screen to obtain a homogeneous fine powder. 0.5 g portions were transferred into 50 mL amber volumetric flasks. Thermal processing was conducted in a dry oven at 180°C for 10, 30, 60, and 90 min, and unheated *C. longa* powder served as the untreated control. After heating, the samples were naturally cooled to ambient temperature. The selected temperature and durations were based on previous findings indicating that curcumin, the predominant bioactive constituent of *C. longa*, undergoes extensive degradation near 180°C within approximately 70 min [8]. The upper limit of 90 min at 180°C was chosen based on the findings of Yun *et al*. [4], who reported the continuous decomposition of curcuminoids and simultaneous accumulation of thermal degradation products under similar conditions.

### Extraction of Thermally Treated Samples

Each thermally processed sample (equivalent to 0.5 g of initial dry powder) was mixed into 50 mL of ethanol and subjected to a 30-minute sonication extraction procedure in an ultrasonic bath (50 kHz, 500 W) at 35°C. The extract was then filtered through a 0.22 μm regenerated-cellulose (RC) membrane filter (Sartorius, Germany). Ten mL aliquots of each filtrate (corresponding to 0.1 g of the original turmeric powder) were concentrated under a gentle nitrogen stream using a heating block maintained at 40°C, freeze-dried, and stored at -80°C until analysis. The dried extracts were reconstituted in either methanol, if destined for HPLC and UHPLC–MS/MS measurements, or dimethyl sulfoxide (DMSO), if destined for anti-inflammatory assays.

### Preparation of Standard and Working Solutions

For HPLC quantification, 1.0 mg/mL standard stock solutions of curcumin, DMC, BDMC, bisacurone, and *ar*-turmerone were prepared in methanol and stored at -20°C. Working solutions were freshly prepared by serially diluting the stock solutions with methanol to obtain concentrations ranging from 0.2 to 25 μg/mL prior to HPLC analysis.

For UHPLC–MS/MS quantification, 1.0 mg/mL standard stock solutions of vanillin, dehydrozingerone, and vanillin-^13^C_6_ were likewise prepared in methanol and stored at -20°C. Working solutions were serially diluted with methanol to yield concentrations between 0.01 and 2 μg/mL immediately before UHPLC–MS/MS analysis. Vanillin-^13^C_6_ at 0.5 μg/mL was used as the internal standard for quantification.

### Quantitative Analysis of Phytochemicals by HPLC

HPLC analysis was performed under the chromatographic conditions described in our previous report [19] using an Agilent 1260 Infinity system equipped with a diode array detector and a Zorbax Plus C18 column (5 μm, 4.6 × 250 mm; Agilent Technologies, USA). Detection wavelengths were set at 424 nm for curcuminoids and 242 nm for bisacurone and *ar*-turmerone.

### Quantitative Analysis of Thermal Degradation Compounds by UHPLC–MS/MS

The quantitative determination of vanillin and dehydrozingerone was conducted using a Thermo Scientific^TM^ Vanquish Flex UHPLC system coupled with a TSQ Altis™ Plus triple quadrupole mass spectrometer (Thermo Fisher Scientific, USA). Chromatographic separation was performed in an Imtakt Unison UK-C18 column (2.0 × 100 μm, 3 μm; Imtakt, Japan) maintained at 30°C. The mobile phases consisted of solvent A (0.1% formic acid in water) and solvent B (0.1% formic acid in acetonitrile), delivered at a flow rate of 0.2 mL/min. In the 11-minute gradient program, solvent B began at 10%, which was held for 1 min, and then linearly increased to 90% over minutes 2–5, maintained at 90% until minute 6, and then returned to 10% at minute 6.1 and allowed to equilibrate until minute 11. The injection volume was 5 μL.

The electrospray ionization (ESI) source parameters included a spray voltage of -3.0 kV (in negative mode for vanillin) or +4.0 kV (in positive mode for dehydrozingerone); a sheath gas of 50 (arbitrary units); an auxiliary gas of 10 a.u.; a sweep gas of 1 a.u.; an ion transfer tube temperature of 300°C; and a vaporizer temperature of 350°C. The mass spectrometer was operated in multiple reaction monitoring (MRM) mode using compound-specific precursor/product ion transitions and optimized collision energies, which are summarized in Table S1. This analytical method, originally developed for vanillin determination, was adapted from Wang *et al*. [20] with modifications to enable the concurrent quantification of dehydrozingerone. Vanillin-^13^C_6_ was employed as the internal standard for both analytes to ensure accurate quantification.

### Cell Culture

RAW 264.7 murine macrophage cells and human bronchial epithelial BEAS-2B cells (Korean Cell Line Bank, Republic of Korea) were cultured in DMEM containing FBS at 10% and penicillin–streptomycin at 1% in a humidified 5% CO_2_ incubator at 37°C. The cells were subcultured every two days to maintain exponential growth.

### Cell Viability

To assess the effects of *C. longa* extract (CLE) on cell viability, RAW 264.7 cells and BEAS-2B cells were seeded into a 96-well plate at 2 × 10^5^ cells/mL and 4 × 10^4^ cells/mL, respectively, and allowed to adhere for 24 h. RAW 264.7 cells were treated with CLE for 24 h to evaluate the cytotoxicity of CLE. For BEAS-2B cells, 1-NP was dissolved in DMSO as a stock solution and diluted in culture medium immediately before use. BEAS-2B cells were pretreated with CLE for 1 h and subsequently exposed to 50 μM 1-NP for 24 h to evaluate the cytoprotection. Cell viability was then measured using the MTT assay. 10 μL of MTT reagent was added to each well, and the plate was incubated for 2 h. Subsequently, 80 μL of the culture medium was removed and 100 μL of DMSO was added to dissolve the formazan crystals. After shaking for 30 min, absorbance was recorded at 570 nm using a microplate reader (Bio-Rad Inc., USA). Data were expressed as a percentage of the untreated control (mean ± SD, n = 3).

### Nitrite Assay

Nitrite accumulation in cell culture supernatants was determined using the Griess assay. RAW 264.7 cells were seeded into a 96-well plate at 37°C in a CO_2_ incubator overnight. Cells were then pretreated with the 90-minute-heated CLE for 1 h and stimulated with LPS (*E. coli* O111:B4, Sigma-Aldrich) for 24 h. After incubation, 100 μL of supernatant was transferred to a new plate, mixed with an equal volume of Griess reagent, 1% sulfanilamide, 0.1% N-(1-naphthyl) ethylenediamine dihydrochloride, and 0.1% phosphoric acid in water, and incubated at room temperature for 10 min. Absorbance was measured at 550 nm using a microplate reader, and the nitrite concentration was calculated using a 0–100 μM concentration curve derived from sodium nitrite standards.

### Western Blot Assay

RAW 264.7 cells were seeded into a 6-well plate and pretreated with 90-minute-heated CLE for 1 h before the cells were exposed to 1 μg/mL of LPS for 24 h. Cells were washed twice with cold phosphate-buffered saline (PBS) and lysed in 80 μL of lysis buffer (Cell Signaling Technologies) on ice for 30 min, with vortexing every 10 min. The lysates were centrifuged at 13,000 × g for 15 min at 4°C, and protein concentrations were quantified using the DC™ Protein Assay Kit (Bio-Rad). Equal amounts of protein (20–30 μg) were separated in a 10% SDS-PAGE gel and transferred to a PVDF membrane (Immobilon?-P; Millipore, USA). Membranes were blocked with 5% skim milk in TBST solution for 1 h, and then incubated overnight at 4°C with antibodies against iNOS (#13120), phospho-p65 (#3033), p65 (#8242), phospho-IκBα (#2859), IκBα (#4814), phospho-IKKα/β (#2697), IKKα (#61294), α/β-tubulin (#2148), PCNA (#2586), p-p38 (#4511), p-JNK (#4668), p-ERK (#4370), Nrf2 (#12721), KEAP1 (#8047), p62 (#39749) (all 1:1000; Cell Signaling Technologies), and β-actin (sc-47778) (Santa Cruz Biotechnology). After washing for 5 min, membranes were incubated with secondary antibodies (1:1000; Thermo Fisher Scientific) in a 5% skim milk solution for 1 h and visualized using Ez West Lumi plus (ATTO, Japan) in a GeneGnome XRQ NPC imaging system (Syngene, UK).

### Cytosol and Nucleus Fraction

Cytosolic and nuclear fractions were isolated using the NE-PER Nuclear and Cytoplasmic Extraction Reagents kit (Thermo Fisher Scientific) following the manufacturer’s instructions. Briefly, Raw 264.7 cells were seeded into a 6 cm dish at 4 × 10^5^ cells/mL, and the plate was incubated for 24 h. Cells were pretreated with the 90-minute-heated CLE for 1 h and then stimulated with 1 μg/mL of LPS for 30 min. The resulting cytoplasmic and nuclear extracts were subjected to western blot analyses to examine p65 translocation, using α/β-tubulin and PCNA as cytosolic and nuclear markers, respectively.

### Immunofluorescence Assay

RAW 264.7 cells were seeded at 4 × 10^5^ cells/mL into 8-well chamber slides (ibidi, GmbH, Germany) and incubated overnight. Cells were pretreated with the 90-minute-heated CLE for 1 h before LPS (1 μg/mL) stimulation for 30 min. The cells were then fixed with 4% paraformaldehyde for 10 min at room temperature, and permeabilized with 100% methanol for 10 min at -20°C. After blocking with 3% Bovine serum albumin for 1 h, the cells were incubated with anti-p65 antibody (1:200; Cell Signaling Technology, USA) overnight at 4°C. The next day, they were washed and incubated with Alexa Fluor 488-conjugated goat anti-Rabbit IgG secondary antibody (1:200; Abcam, UK) for 1 h. Nuclei were counterstained with DAPI, and coverslips were mounted with VECTASHIELD antifade medium (Vector Laboratories, USA). Images were acquired using a fluorescence microscope (Leica Microsystems, Germany).

## Results

### Thermally Induced Variations in the Bioactive Constituents and Degradation Metabolites of *Curcuma longa* (*ulgeum*) Extract

The quantitative changes in the major phytochemicals of Korean-cultivated *C. longa* (*ulgeum*) subjected to thermal treatment at 180°C for 10, 30, 60, and 90 min are summarized in Table 1. Prolonged heating resulted in a substantial reduction in native curcuminoids. After 90 min, the concentration of curcumin (2572.6 mg/kg in untreated CLE vs. 37.4 mg/kg in 90-minute-heated CLE), BDMC (236.9 mg/kg vs. 16.0 mg/kg), and DMC (600.7 mg/kg vs. 17.8 mg/kg) were lower than those in the unheated control. Bisacurone also showed substantial thermal degradation, decreasing from 251.9 mg/kg to 1.3 mg/kg. Although *ar*-Turmerone declined from 2322.2 mg/kg to 1560.2 mg/kg under the same conditions, it was more thermally stable than the curcuminoids and bisacurone. In contrast, Vanillin (13.1 mg/kg vs. 42.6 mg/kg) and DHZ (3.1 mg/kg vs. 13.6 mg/kg) increased after 90 min. Overall, heating substantially altered the chemical composition of the *ulgeum* extract, resulting in a pronounced decline in native curcuminoids and the concomitant formation of low-molecular-weight degradation products.

### CLE Attenuates 1-Nitropyrene-Induced Cell Toxicity in BEAS-2B Cells

Human bronchial epithelial BEAS-2B cells were used to assess the cytoprotective effects of untreated CLE and CLEs heat-treated for 10, 30, 60, and 90 min. Based on previous finding that 1-NP induces cell death [17], we used 1-NP as the cytotoxicity model. Among the tested conditions, the 60-minute-heat-treated CLE significantly attenuated 1-NP-induced cell death, exhibiting greater protective efficacy than the untreated or 0-, and 90-minute-treated extracts (Fig. 1A). Based on these results, the 60-minute-heat-treated CLE was selected and treatment at concentrations ranging from 6.25 to 50 μg/mL significantly suppressed 1-NP-induced cell death in a dose-dependent manner (Fig. 1B).

### CLE inhibits LPS-induced nitrite production and suppresses iNOS and COX-2 expression in RAW 264.7 cells

Minimizing the aberrant activation of inflammatory pathways is critical for preventing excessive immune responses and preserving physiological homeostasis [21]. RAW 264.7 cells were used to evaluate the anti-inflammatory effects of untreated CLE and CLEs heat-treated for 10, 30, 60, and 90 min. Each extract, applied at concentrations of 10, 20, and 40 μg/mL led to a dose-dependent suppression of LPS-induced nitrite (NO) production in RAW 264.7 cells (Fig. 2A). To evaluate the factors involved in inflammation, we examined the expression of iNOS in RAW 264.7 cells exposed to the CLEs. All heat-treated extracts suppressed LPS-induced iNOS expression in RAW 264.7 cells (Fig. 2B), and the 90-minute-heat-treated CLE was chosen for further analysis due to its inhibitory activity. The expression levels of iNOS and COX-2 were analyzed in LPS-exposed cells pretreated with 90-minute-heat-treated CLE at concentrations of 12.5, 25, and 50 μg/mL, and LPS-induced iNOS expression was inhibited in a dose-dependent manner, while COX-2 expression was significantly attenuated at 50 μg/mL (Fig. 2C-2E). The 90-minute-heat-treated cytotoxicity at 100 and 200 μg/mL concentrations (Fig. 2F). Together, these results demonstrate that heating does not diminish the anti-inflammatory capacity of CLE, which continues to modulate NO, iNOS, and COX-2 expressions in RAW 264.7 Cells.

### CLE Reduces LPS-Induced NF-κB Signaling in RAW 264.7 Cells

The expression of iNOS and COX-2 is known to be regulated by the NF-κB signaling pathway [22]. To determine whether 90-minute-heat-treated CLE modulates LPS-induced NF-κB activation, phosphorylation of the NF-κB subunit p65 was examined. The CLE markedly reduced LPS-induced p65 phosphorylation at 50 μg/mL in both whole-cell lysates and nuclear fractions (Fig. 3A and 3B). In addition, IKK phosphorylation was decreased at 50 μg/mL, accompanied by increased IκBα levels at 50 μg/mL (Fig. 3C). In contrast, the CLE did not affect the LPS-induced phosphorylation of mitogen-activated protein kinases JNK1/2, p38, and ERK1/2 (Fig. 3D).

### CLE Ameliorates LPS-Induced ROS Generation and Activates Nrf2 Activation in RAW 264.7 Cells

Lipopolysaccharide-induced ROS generation was significantly reduced in a concentration-dependent manner by CLEs in all heat-treatment groups, including the non-heated extract (Fig. 4A). Representative DCF-DA fluorescence imaging revealed that ROS levels were reduced in cells exposed to the 90-min-heat-treated CLE compared with the LPS-only cells (Fig. 4B). To further examine whether this reduction was associated with the activation of antioxidant signaling, cytosolic and nuclear fractions were isolated to assess Nrf2 nuclear translocation. In the cytosolic fraction, keap1 expression was not altered by either LPS or CLE treatment, whereas p62 expression was increased by LPS but showed no additional modulation when a CLE pretreatment was included (Fig. 4C). Treatment with 90-min-heat-treated CLE significantly increased nuclear Nrf2 levels at 20 and 40 μg/mL (Fig. 4D and 4E). These results demonstrate that CLE continues promoting Nrf2 nuclear translocation even after thermal processing.

## Discussion

This study investigated how heating alters the major constituents of Korean-cultivated *C. longa* L. rhizome and evaluated its impact on cytoprotective, anti-oxidant, and anti-inflammatory activities. Because *C. longa* is commonly consumed after heat processing, understanding the thermal stability of its bioactive components is essential. In our previous study, we characterized the heat-induced metabolite profile of Korean *ulgeum* and Indian turmeric methanol extracts using UHPLC-HRMS and GC-MS [4]. *Ulgeum* was found to contain distinct terpenoids, including β-elemenone, curcumenone, and germacrone, that were absent in Indian turmeric, highlighting a clear metabolic divergence despite their shared botanical origin. These metabolic differences may, thus, reflect environmental and climatic adaptations rather than species-level variation. Based on these findings, we hypothesized that heat treatments may modulate *C. longa*’s bioactive potential rather than uniformly diminishing its efficacy. To address this hypothesis in a physiologically relevant context, we employed a 1-NP-induced lung epithelial toxicity model and an LPS-induced macrophage inflammation model, as the functional integrity of epithelial cells and macrophages is critical for maintaining pulmonary homeostasis.

Consistent with our previous reports [4], curcuminoids were highly susceptible to thermal degradation, exhibiting rapid losses during heating at 180°C. In this study, curcumin, bisdemethoxycurcumin, and demethoxycurcumin decreased by approximately 74–79% within 30 min of heating and by more than 93% after 90 min. This pronounced loss early in the treatment reflects the intrinsic instability of curcuminoids, which readily undergo oxidative degradation and molecular rearrangement through β-diketone cleavage and subsequent intramolecular reactions [5, 8, 23]. Curcuminoids are known to be extremely sensitive to thermal and photolytic degradation, undergoing rapid decomposition during food preparation [23, 24]. In contrast, sesquiterpenoids demonstrated greater thermal stability than curcuminoids. After 30 min of heating, *ar*-turmerone and bisacurone decreased by approximately 21% and 46%, respectively, indicating a markedly slower degradation rate than that of curcuminoids. This enhanced stability can be attributed to the absence of the conjugated β-diketone moiety that renders curcuminoids particularly vulnerable to thermal and oxidative stress. Although these sesquiterpenoids exhibited greater thermal stability during the early stages of heating, their degradation patterns diverged markedly with prolonged exposure. After 90 min at 180°C, *ar*-turmerone had decreased by approximately 33%, whereas bisacurone exhibited a nearly complete loss of 99%. Although both contain an α, β-unsaturated ketone moiety, differences in their surrounding structural environments modulate their susceptibility to heat-induced degradation. *Ar*-turmerone possesses an aromatic ring lacking hydroxyl group [25], which may limit oxidative and rearrangement reactions. In contrast, bisacurone contains an aliphatic backbone with multiple oxygenated functional groups [26], a structural feature that is likely associated with increased chemical reactivity and promote thermal decomposition. These observations highlight how subtle structural features can govern the long-term stability of these sesquiterpenoids under thermal processing.

Furthermore, trace levels of vanillin and dehydrozingerone were formed during curcuminoid degradation. Although their absolute concentrations were low, these thermally derived phenolic metabolites have been reported to exert potent radical-scavenging and anti-inflammatory activities [8, 20]. The accumulation of these degradation products, along with the stability of sesquiterpenoids, may have contributed to maintaining the functional integrity of the heat-treated extracts. Similar observations have been reported by Esatbeyoglu *et al*., Oh *et al*., and Yun *et al*. [4, 8, 13], who observed that turmerone-type compounds and curcumin-derived metabolites maintained notable antioxidant and anti-inflammatory effects even after thermal exposure. Importantly, the substantial decrease in total curcuminoid content far exceeded the increase in identifiable phenolic degradation products, indicating that curcuminoids were not solely converted into vanillin and dehydrozingerone. This discrepancy suggests the involvement of additional degradation routes, including polymerization and volatilization, and/or the formation of unidentified intermediates. Collectively, these findings indicate that the biological efficacy of thermally processed *C. longa* cannot be attributed exclusively to residual curcuminoids but instead reflects the composite effect of multiple interacting constituents generated or retained during heat processing.

At the cellular level, heat-treated CLEs exhibited distinct biological effects depending on heating duration, while overall functional efficacy was preserved. Among the tested heating durations, CLEs heated for 60 min conferred the most pronounced protective effect against 1-NP-induced epithelial cell death, suggesting an optimal balance between compound degradation and retention. Moreover, different heat treatments influenced inflammatory mediator production differently. While all CLEs significantly attenuated both NO and ROS generation, NO levels were most effectively reduced by the 10-minute-heat-treated extract, whereas ROS suppression was most prominent following treatment with the 10- and 90-minute-heat-treated CLEs. Importantly, anti-inflammatory and antioxidant activities were largely preserved, even after 90 min of heating, indicating that prolonged thermal exposure does not destroy the CLE’s bioactivity. Based on our previous study, *ar*-turmerone has been shown to mitigate ROS production with high efficacy, whereas vanillin and dehydrozingerone exhibit comparatively weaker antioxidant effects [27]. These compound-specific differences in intrinsic bioactivity may partly explain the distinct antioxidant and anti-inflammatory responses observed when applying heat-treated CLEs, suggesting they are a function of evolving compositional changes associated with heating duration. Taken together, these observations underscore the need for further studies integrating comprehensive metabolite profiling with kinetic analyses to clarify which metabolites persist following thermal processing and to determine how individual constituents contribute to the preservation of bioactivity in heat-processed Korean *C. longa* extract.

Given that pretreatment with CLEs heated for 90 min suppressed NO production in LPS-stimulated RAW264.7 macrophages, we examined the associated inflammatory signaling pathways. The inhibitory effect of heat-treated CLE was linked to the attenuation of NF-κB activation, whereas MAPK signaling pathways remained unaffected, suggesting that the CLE-mediated suppression of NO production occurs primarily through the selective modulation of NF-κB signaling. Importantly, the antioxidant efficacy of CLEs was maintained across the entire range of heating times, from 0 (untreated) to 90 min, as indicated by the sustained suppression of ROS production. Cellular ROS levels are tightly regulated by transcription factor Nrf2, which coordinates antioxidant defenses by inducing cytoprotective protein expression [28, 29]. Although p62/SQSTM1 has been reported to disrupt Keap1–Nrf2 interactions and promote Nrf2 stabilization [30], the present findings indicate that CLE-induced Nrf2 activation occurs without detectable modulation of cytosolic Keap1 or p62 expression. Together, these observations support a model in which, even after prolonged thermal processing, CLE enhances antioxidant capacity by promoting Nrf2 nuclear translocation. The concurrent attenuation of NF-κB signaling and activation of Nrf2-dependent antioxidant responses suggest a coordinated regulation of inflammatory and redox pathways that is preserved through thermal processing.

## Conclusion

This study demonstrates that high-temperature processing profoundly alters the chemical composition of Korean-cultivated *C. longa* (*Ulgeum*) extracts while preserving their anti-inflammatory and antioxidant bioactivities. Although curcuminoids were highly susceptible to thermal degradation, substantial biological efficacy was retained by the CLEs following heat treatment, even after prolonged exposure at 180°C. The retention of bioactivity despite substantial curcuminoid degradation suggest that the observation effects may be associated with broader heat-induced changes in extract composition, including the persistence of relatively thermostable sesquiterpenoids and the formation of phenolic metabolites, rather than with curcuminoid content alone. In cell culture models, heat-treated extracts suppressed inflammatory responses by attenuating NF-κB signaling while concurrently increasing nuclear Nrf2 levels without affecting MAPK pathways. Importantly, CLEs subjected to intermediate-duration heat treatments conferred the strongest cytoprotective effects against toxicant-induced epithelial injury, demonstrating that thermal processing does not uniformly diminish bioactivity, but can instead shape functional outcomes by modulating extract composition. Collectively, these findings suggest that the bioactivity of *Ulgeum* may be associated with the coordinated regulation of inflammation and oxidative stress, underscoring the functional robustness of heat-*processed* *C. longa* as a dietary botanical.

## Supplemental Materials

Supplementary data for this paper are available on-line only at http://jmb.or.kr.



## Figures and Tables

**Fig. 1:**
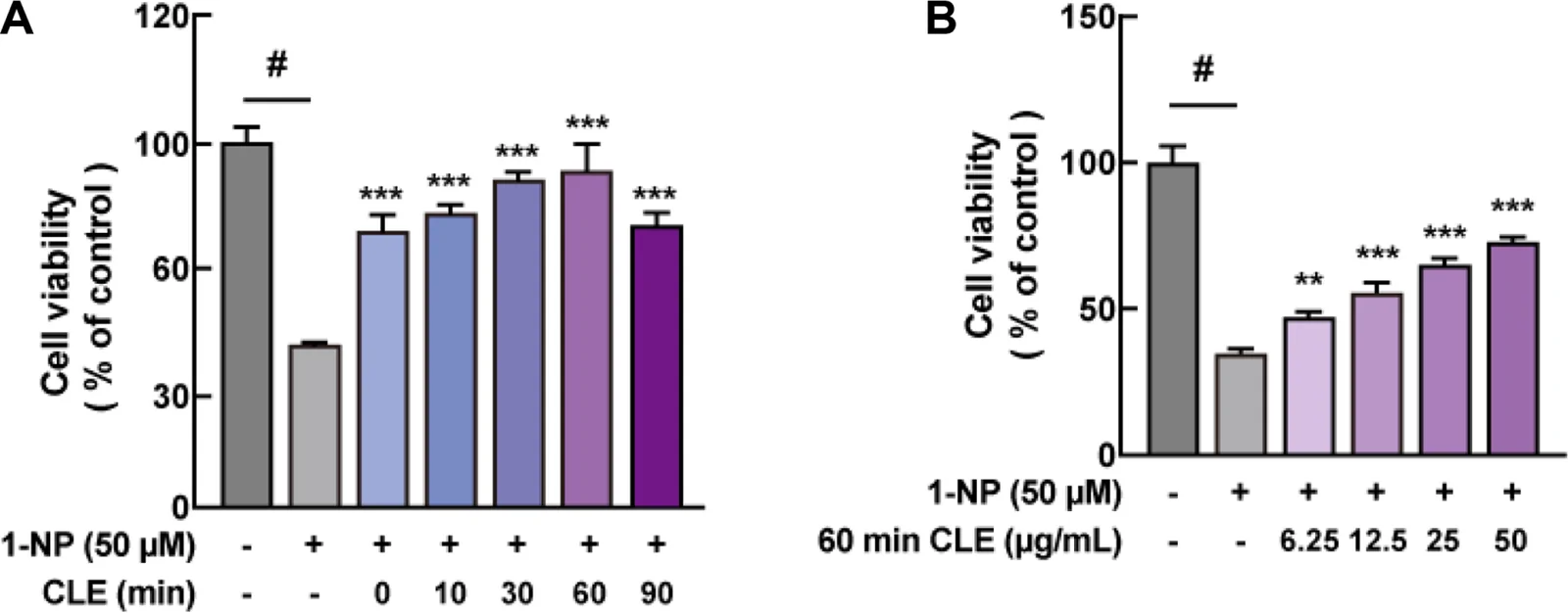
Effect of heat-treated CLE on 1-nitropyrene-induced cytotoxicity in BEAS-2B cells. (**A**) Cell viability of BEAS-2B bronchial epithelial cells was assessed using MTT assay after pretreatment with heat-treated *Curcuma longa* extracts (CLEs; 0, 10, 30, 60, and 90 min, 50 μg/mL), followed by exposure to 1-NP (50 μM) for 24 h. (**B**) Dose-dependent effects of the 60-min heat-treated CLE (6.25–50 μg/mL) against 1-NP (50 μM)-induced cytotoxicity in BEAS-2B cells. Data are expressed as mean ± SD (n = 3). Statistical significance was determined using one-way ANOVA followed by Dunnett’s multiple comparison test. #*p* < 0.05 vs. untreated control; **p < 0.01 and ***p < 0.001 vs. 1-NP alone.

**Fig. 2:**
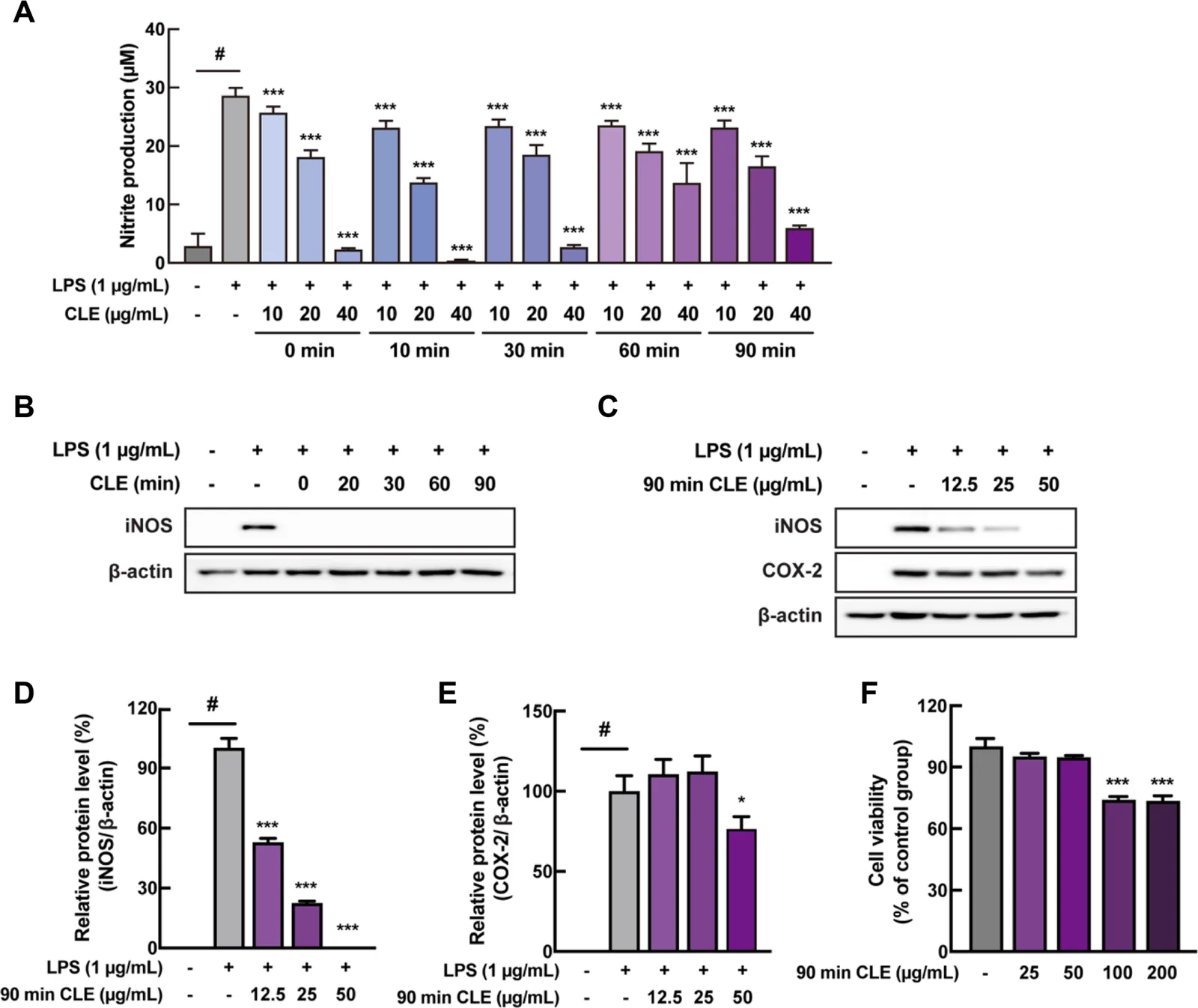
Heat-treated CLE suppresses LPS-induced inflammatory mediators in RAW 264.7 macrophages. (**A**) Effects of CLEs subjected to heat treatment for different durations (0, 10, 30, 60, and 90 min; 10–40 μg/mL) on lipopolysaccharide (LPS)-induced nitrite production in RAW 264.7 macrophages. Cells were pretreated with CLE for 1 h and then stimulated with LPS (1 μg/mL) for 24 h. Nitrite levels in culture supernatants were quantified using the Griess assay. (**B**) Representative immunoblot images showing iNOS expression in RAW 264.7 cells pretreated with CLEs heat-treated for different durations (0−90 min), followed by LPS stimulation (1 μg/mL). (**C**) Representative immunoblot images showing iNOS and COX-2 expression in RAW 264.7 cells pretreated with 90 min heat-treated CLE (12.5–50 μg/mL), followed by LPS (1 μg/mL). β-actin was used as a loading control. (**D, E**) Quantification of iNOS and COX-2 expression normalized to β-actin. (**F**) Cytotoxicity of 90 min heat-treated CLE in RAW 264.7 cells. Cell viability was assessed after treatment with the 90 min heat-treated CLE (25–200 μg/mL). Data are expressed as mean ± SD (n = 3). Statistical significance was determined using one-way ANOVA followed by Dunnett’s multiple comparison test. #*p* < 0.05 vs. untreated control; *p < 0.05 and ***p < 0.001 vs. LPS alone.

**Fig. 3:**
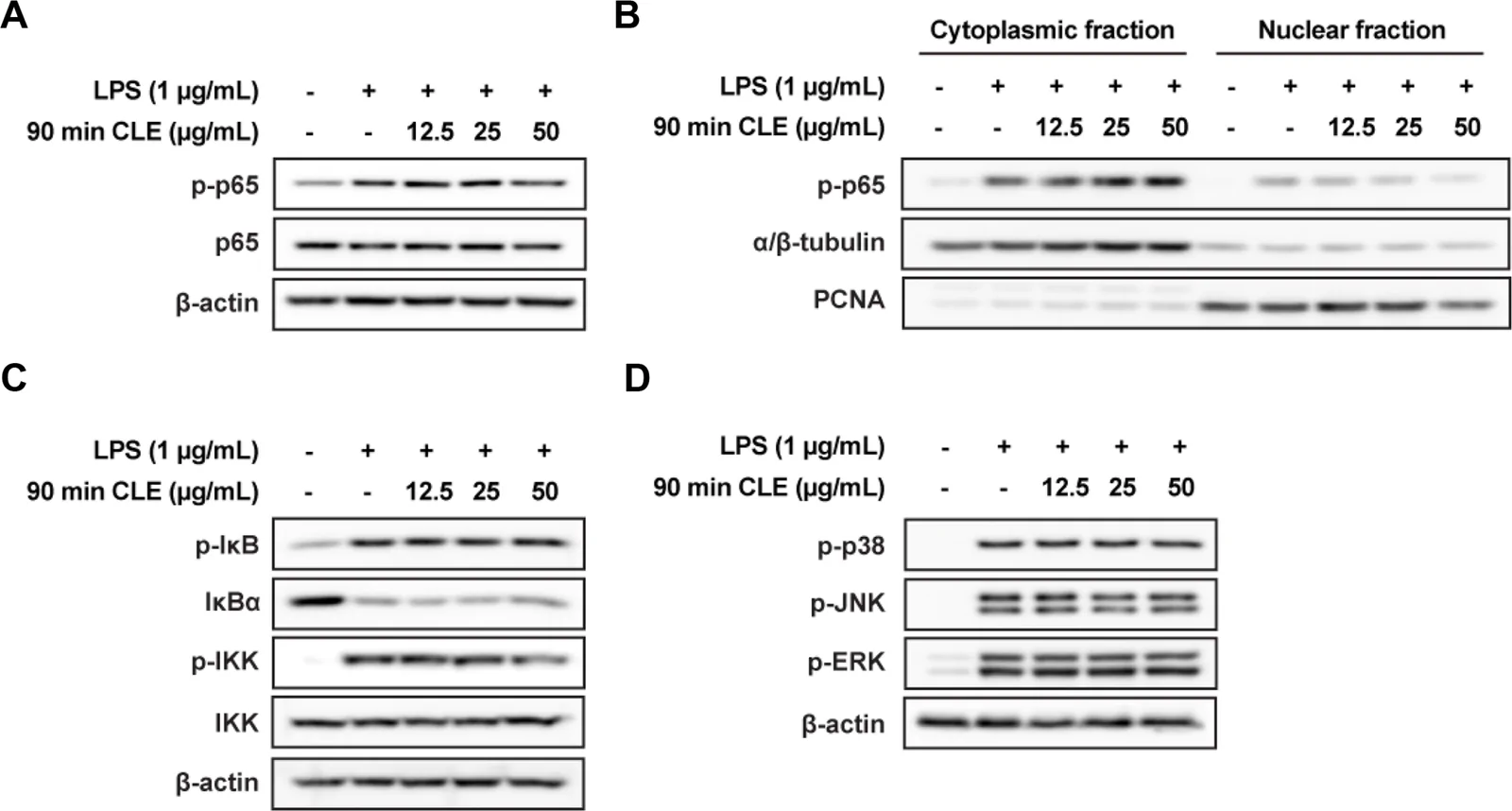
Heat-treated CLE suppresses LPS-induced NF-κB activation in RAW 264.7 macrophages. (**A**) Representative immunoblot images showing phosphorylation of NF-κB p65 and total p65 in RAW 264.7 cells pretreated with the 90 min heat-treated CLE (12.5–50 μg/mL), followed by LPS stimulation (1 μg/mL). α/β-tubulin and PCNA were used as markers for cytosolic and nuclear fractions, respectively. (**C**) Immunoblot analysis of IκBα phosphorylation (p- IκBα), total IκBα, phosphorylation of IKK (p-IKK), and total IKK in RAW 264.7 cells pretreated with the 90 min heat-treated CLE (12.5–50 μg/mL), followed by LPS stimulation (1 μg/mL). β-actin was used as a loading control. (**D**) Immunoblot analysis of MAPK signaling, including phosphorylation of p38 (p-p38), JNK (p-JNK), and ERK (p-ERK).

**Fig. 4:**
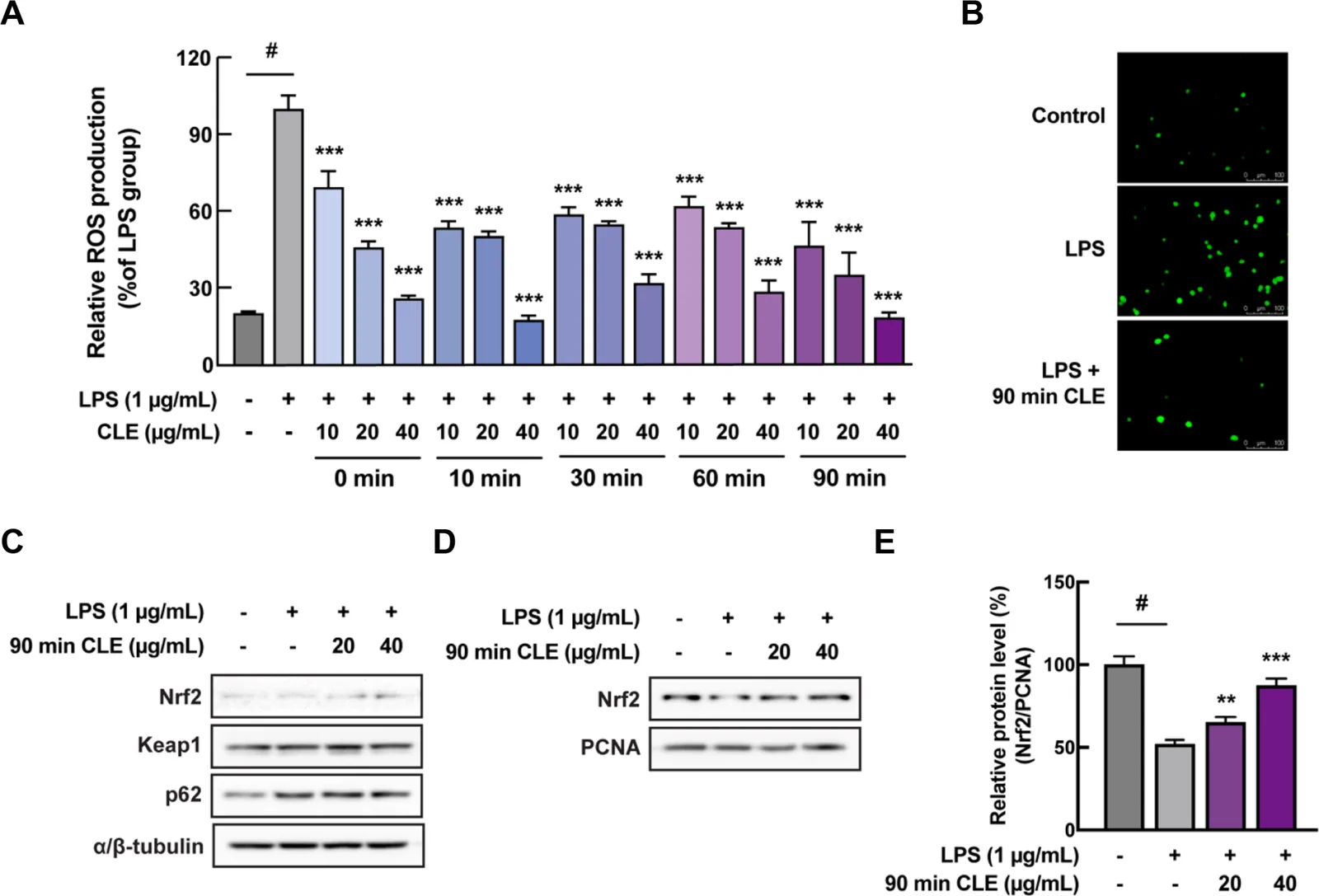
Heat-treated CLE suppresses LPS-induced ROS generation and activates Nrf2-mediated antioxidant responses in RAW 264.7 macrophages. (**A**) Intracellular ROS levels were assessed using DCF-DA fluorescence staining in RAW 264.7 cells treated with LPS (1 μg/mL) in the presence or absence of the 90 min heat-treated CLE (10–40 μg/mL). (**B**) Representative DCF-DA fluorescence images showing intracellular ROS levels. Scale bar = 100 μm. (**C**) Immunoblot analysis of cytosolic Keap1, p62/SQSTM1, and Nrf2 expression in RAW 264.7 cells treated with LPS (1 μg/mL) in the presence or absence of the 90 min heat-treated CLE (20 and 40 μg/mL). α/β-tubulin was used as markers for cytosolic fraction. (**D**) Immunoblot analysis of nuclear Nrf2 expression under the same conditions. PCNA was used as markers for nuclear fraction. (**E**) Quantification of Nrf2 levels in nuclear fraction. Data are expressed as mean ± SD (n = 3). Statistical significance was determined using one-way ANOVA followed by Dunnett’s multiple comparison test. #*p* < 0.05 vs. untreated control; **p < 0.01 and ***p < 0.001 vs. LPS alone.

**Table 1:** Quantitative changes in the major bioactive compounds of *Curcuma longa* (*ulgeum*) extracts during 180°C heat treatments of varying length.

Compound group	Compound (mg/kg)	0 min	10 min	30 min	60 min	90 min
Curcuminoids	BDMC	236.9 ± 2.9^a^	151.4 ± 1.4^b^	48.7 ± 0.4^c^	19.8 ± 0.3^d^	16.0 ± 0.3^d^
	DMC	600.7 ± 9.0^a^	430.7 ± 3.1^b^	133.2 ± 0.2^c^	32.6 ± 0.4^d^	17.8 ± 0.4^d^
	Curcumin	2572.6 ± 36.5^a^	2002.8 ± 14.3^b^	676.9 ± 2.4^c^	131.7 ± 0.4^d^	37.4 ± 0.5^e^
Sesquiterpenoids	*ar*-Turmerone	2322.2 ± 33.6^a^	2138.9 ± 12.3^b^	1837.3 ± 1.2^c^	1757.0 ± 1.6^c^	1560.2 ± 5.6^d^
	Bisacurone	251.9 ± 0.6^a^	248.1 ± 1.3^a^	136.4 ± 0.9^b^	36.0 ± 0.7^c^	1.3 ± 0.0^d^
Thermal degradation products	Vanillin	13.1 ± 0.3^a^	19.9 ± 1.8^b^	34.3 ± 0.3^c^	43.9 ± 0.8^d^	42.6 ± 0.1^d^
	DHZ	3.1 ± 0.1^a^	5.0 ± 0.3^b^	17.9 ± 0.3^c^	18.7 ± 0.3^c^	13.6 ± 0.5^d^

Values represent the mean ± the standard deviation (*n* = 3).

Different superscript letters (a–e) within a row indicate significant differences among heating times (*p* < 0.05).

Abbreviations include BDMC (bisdemethoxycurcumin), DMC (demethoxycurcumin), and DHZ (dehydrozingerone).
