# Supplementary material for: Bioactivity of Heat-Processed Korean Curcuma longa is Preserved Despite Extensive Curcuminoid Degradation
Source: J Microbiol Biotechnol. 2026 Sep 18;36:e2606010. doi: 10.4014/jmb.2606.06010 (PMC13696954; doi:10.4014/jmb.2606.06010)
Supplement: Supplementary file 1 [file jmb-36-e2606010-supple.pdf]

J. Microbiol. Biotechnol. 2026. 36: e2606010
https://doi.org/10.4014/jmb.2606.06010

Table S1. UHPLC–MS/MS MRM conditions for the quantitative determination of vanillin and dehydrozingerone.

Compounds	Molecular formula	Polarity [ESI±]	Precursor ion (m/z)	Product ions (m/z)	Collision energy (V)	RF lens (V)	Retention time (min)
Vanillin	$C_8H_8O_3$	$[M-H]^-$	151	136<br>92<br>108	13<br>20<br>22	85	5.3
Dehydrozingerone	$C_{11}H_{12}O_3$	$[M+H]^+$	193	115<br>175<br>143	19<br>13<br>17	114	5.7
Vanillin $^{13}C_6$ (Internal Standard)	$[^{13}C_6]C_8H_8O_3$	$[M+H]^+$	159	99	15	83	5.3
